# Prenatal famine exposure and adult health outcomes: an epigenetic link

**DOI:** 10.1093/eep/dvab013

**Published:** 2021-11-24

**Authors:** Alexander Vaiserman, Oleh Lushchak

**Affiliations:** Laboratory of Epigenetics, D.F. Chebotarev Institute of Gerontology, NAMS, 67 Vyshgorodska St., Kyiv 04114, Ukraine; Department of Biochemistry and Biotechnology, Vasyl Stefanyk Precarpathian National University, 57 Shevchenka St., Ivano-Frankivsk 76018, Ukraine; Research and Development University, 13A Shota Rustaveli St., Ivano-Frankivsk 76000, Ukraine

**Keywords:** prenatal famine exposure, natural experiment, DOHaD concept, adult disease, epigenetic change, DNA methylation

## Abstract

Numerous human chronic pathological conditions depend on epigenetic modifications induced by environmental triggers throughout sensitive stages early in development. Developmental malnutrition is regarded as one of the most important risk factors in these processes. We present an overview of studies that the initiation and progression of many diseases are largely dependent on persisting epigenetic dysregulation caused by environmental insults early in life. For particular disorders, candidate genes were identified that underlie these associations. The current study assessed the most convincing evidence for the epigenetic link between developmental malnutrition and adult-life disease in the human population. These findings were obtained from quasi-experimental studies (so-called ‘natural experiments’), i.e. naturally occurring environmental conditions in which certain subsets of the population have differing levels of exposure to a supposed causal factor. Most of this evidence was derived on the DNA methylation level. We discussed DNA methylation as a key player in epigenetic modifications that can be inherited through multiple cell divisions. In this Perspective article, an overview of the quasi-experimental epidemiological evidence for the role of epigenetic mechanisms in the developmental programming by early-life undernutrition is provided.

## Introduction

Compelling evidence indicates that developmental exposure to adverse environmental conditions can programme the risk for chronic disorders in adult life. According to the Developmental Origins of Health and Disease (DOHaD) concept that was proposed to explain this phenomenon, such exposure may reprogramme an individual for immediate adaptation to potentially stressful postnatal experiences but affect the risk for adult-life pathological conditions such as cardiometabolic and neurodegenerative disorders and cancer, as well as longevity [1, 2]. Developmental malnutrition is regarded as one of the most important risk factors in these processes [3, 4]. In particular, prenatal nutritional insufficiency can cause developmental adaptations, which prepare the foetus for survival in conditions of postnatal nutritional scarcity. However, if offspring does not face food scarcity in postnatal life, such a discrepancy between predicted and actual postnatal environments can cause cardiovascular and metabolic disorders in adulthood. The mechanisms underlying the developmental nutritional programming phenomenon are being actively studied. Recently, consistent evidence from experimental and epidemiological studies was obtained demonstrating the key role of epigenetic factors, including DNA methylation, histone modification and non-coding RNA-associated gene activation/silencing in mediating these effects [5, 6]. Over several recent years, evidence is obtained from epigenome-wide association studies that initiation and progression of many diseases are largely dependent on persisting epigenetic dysregulation caused by environmental insults early in life. For several disorders, candidate genes were identified that underlie these associations.

In human populations, most convincing evidence for the epigenetic link between developmental malnutrition and adult-life disease was obtained from quasi-experimental studies (so-called ‘natural experiments’), i.e. naturally occurring environmental conditions in which certain subsets of the population have differing levels of exposure to a supposed causal factor [7]. Most of this evidence was derived on the DNA methylation level. Exploring processes at this level is all the more important that DNA methylation undergoes the most important modification during particular developmental stages such as gametogenesis and embryogenesis; importantly, during these stages, epigenome is most susceptible to adverse environmental cues [8]. Another important point is that developmentally established epigenetic marks may be stably maintained through subsequent somatic cell divisions and persisted across the entire life course. In mammals, including humans, windows of developmental epigenetic plasticity were shown to extend from the preconception period until early childhood (the ‘first 8000 days of life’ concept) [9], and environmental exposures during this period can contribute to tuning the organism’s gene expression programme (as well as so-called ‘epigenetic clock’) [10].

In this opinion article, an overview of the epidemiological evidence obtained from quasi-experimental studies for the developmental programming by early-life malnutrition and for the role of epigenetic mechanisms in these processes is provided.

## DOHaD Paradigm: Research Findings and Conceptual Insights from Human Studies

Multiple findings from animal models indicate that inadequate nutrition during critical developmental periods can cause permanent structural and/or functional impairments of various tissues and organs and lead to long-term adverse health consequences [11]. These findings have been also repeatedly confirmed in epidemiological studies. The pioneering ‘fetal origins of adult disease’ hypothesis, which was an attempt to explain the nutritional programming phenomenon, has been based on epidemiological observations that prenatal undernutrition-induced fetal growth retardation and associated small size at birth are related to increased risks for type 2 diabetes (T2D) and cardiovascular disorders in adulthood [12, 13]. Studies of prenatal famine and later outcomes in the Netherlands began with the work of Stein and colleagues in 1972 who later demonstrated reduced postpartum maternal weight, birth weight, placental weight, length at birth and head circumference at birth as a result of prenatal exposure to the Dutch famine of 1944–45 [14]. Since the pioneering works by Barker and colleagues [12], it has been repeatedly shown that accelerated [catch-up] postnatal growth, by which babies born with low birth weight reach or even exceed normal body weight in their subsequent life, is associated with high risks of adiposity, insulin resistance, T2D and cardiovascular diseases later in life [15]. Based upon initial findings, it has been suggested that a linear inverse relationship exists among birth weight and subsequent risk for T2D [16]. However, the original study found no relation of prenatal exposure to famine and cognitive performance at age 18 in males but showed increased obesity in males after exposure to famine in early/mid-gestation [17]. Yet, the increased risk for obesity and metabolic disorders in more recent studies was among women [18]. More recent meta-analyses, however, have demonstrated that association between birth weight and T2D is rather U-shaped, and high birth weight is associated with high risk for T2D to the same extent as low birth weight [19]. Subsequently, multidirectional relationships between birth weight and different adult disorders have been repeatedly reported in epidemiological studies. For example, in a Danish cohort, the association between birth weight and cancer risk was linear, whereas the association with cardiovascular diseases was U-shaped, and elevated risk of premature death was found in individuals born with either lower or higher weights [20]. Therefore, birth weight is currently considered as an inappropriate proxy for *in utero* etiologic determinants, and present-day epidemiologic studies tend to directly examine the effects of certain intrauterine factors on postnatal health outcomes, irrespective of birth weight [21].

Presently, quasi-experimental (natural experiment-based) design is generally regarded as the most reliable approach for determining cause–effect relationships between early-life exposures and adult-life health outcomes. Famine (both natural and manmade) has apparent eligible features in order to be applied as a natural experiment. The studies we review have all been observational. Moreover, it should be taken into account that the effects of social, economic and family conditions on adult health outcomes contribute to the same extent as nutrition itself. Currently, long-term health outcomes of prenatal exposure to the Dutch famine, which affected the western Netherlands during the German occupation from November 1944 to May 1945, are studied most thoroughly in quasi-experimental setups. Among these adverse outcomes, there are obesity, T2D, and also multiple other cardiometabolic and mental disorders [22, 23]. Furthermore, in women [but not men] exposed to the Dutch famine in early gestation, higher cardiovascular and cancer mortality risks in adulthood were observed compared to unexposed ones [24]. Consistent findings have been also reported in studying other famines, such as the Ukrainian famine of 1932–33 [25] and the Chinese famine of 1959–61 [26, 27]. We emphasize on different lengths, severity, and social context of the famines discussed in the current study. Our main interest was the involvement of epigenetic mechanisms in the long-term consequences of exposure to famine on specific adult health outcomes. Considering different contexts of the discussed famines and great variations in the quality of the studies, many questions on programming still remain unanswered.

## Epigenetic Memory of Prenatal Famine Exposure

Convincing evidence indicates that epigenetic mechanisms such as DNA methylation can mediate the association between prenatal famine exposure and adult-life health outcomes. Most of this evidence was obtained from the Dutch famine birth cohort, although no significant association between prenatal exposure to the Dutch famine and global DNA methylation in adulthood was reported [28]. However, certain gene-specific epigenetic modifications were found to be associated with this exposure in several studies. Hence, global DNA methylation is not informative but could guide further targeted research along specific lines into biological mechanisms. Focusing on selected regions in the genome that may be differentially methylated in response to changes in early-life exposures could predict adult health outcomes [28]. For instance, periconceptional exposure to the Dutch famine has been associated with reduced methylation levels of the insulin-like growth factor 2 (*IGF2*) gene playing a fundamental role in growth and development in exposed subjects aged ∼sixty years compared with their non-exposed siblings [29] (for more specific details, see Table 1). Subsequently, differences in DNA methylation levels between exposed and unexposed siblings were observed for several other genes implicated in growth and metabolic regulation, including *IL10, INSIGF, LEP, MEG3, ABCA1* and *GNASAS* [30]. More recently, genome-wide changes in adult profile of whole-blood DNA methylation were found in individuals prenatally exposed to the Dutch famine; early gestation was shown to be a critical time window for inducing these changes [31]. Importantly, changes in DNA methylation were shown to mediate the link between prenatal exposure to the Dutch famine and metabolic disorders in adulthood [32]. More specifically, such a link was obtained for adult body mass index (BMI) and triglyceride (TG) levels but not for glucose levels. Changes in DNA methylation at *PIM3*, a gene involved in energy metabolism, mediated about 13% of the association between prenatal famine exposure and BMI. DNA methylation at six cytosine-phosphate-guanine dinucleotides (CpGs), including *TXNIP* affecting β-cell function and *ABCG1* influencing lipid metabolism, together mediated about 80% of the association between prenatal famine exposure and serum TG level. Analyses restricted to those persons who were exposed to famine during early gestation identified additional CpGs mediating the relationship with TG near *PFKFB3* (glycolysis) and *METTL8* (adipogenesis). Moreover, in a genome-wide analysis of DNA methylation, differentially methylated regions were found to extend along pathways related to growth and metabolism in subjects periconceptionally exposed to the Dutch famine [33]. Furthermore, the effects of periconceptional exposure to the Dutch famine on DNA methylation at the imprinted *IGF2/H19* region were shown to be dependent on single-nucleotide polymorphisms in the exposed population [34]. Early [but not mid or late] gestation has been identified as a critical period for inducing these changes, and genes implicated in growth, development and metabolism have been demonstrated to be most substantially involved in these processes [34]. However, in investigating the methylation status of proximal promoter regions of four genes associated with markers of metabolic and cardiovascular disorders, such as glucocorticoid receptor, peroxisome proliferator-activated receptor gamma, lipoprotein lipase and phosphatidylinositol 3 kinase p85, no differences in methylation levels of these promoters were observed between individuals exposed to Dutch famine *in utero* and unexposed controls [35]. It is worth noting that several epidemiological methodological papers have shown that under a number of circumstances mediation findings may produce flawed conclusions. However, for the investigation of the molecular mechanisms involved in disease causation and for measuring the impact of public health interventions the study of mediation is necessary for epidemiological studies. The assessment of mediation can be the main aim of the study, whereas often the goal is to estimate the general effect, although exploratory mediation analyses are also conducted [36].

**Table 1:** Overview of epigenetic modifications in adult whole blood induced by early-life famine exposure

Country	Sample, *N*	Exposure time	Age at detection	Gene/element	Epigenetic outcome	Function/pathway	Ref(s).
The Netherlands	60 sibships 62 sibships	Periconception and late gestation	57–59 y	*IGF2*	Decreased methylation	Growth and development	[29]
	60 sibships 62 sibships	Periconception and late gestation	57–59 y	*IL10, LEP, MEG, ABCA1, GNASAS* and *INSIGF*	Hypermethylation Hypomethylation	Growth and metabolic disease	[30]
	422 exposed; 463 sibling controls	Prenatal	∼59 y	MWAS	Differential DNA methylation	Growth, development and metabolism	[31]
	422 exposed subjects; 463 sibling controls	Prenatal	60+	*PIM3, TXNIP, ABCG1, PFKFB3* and *METTL8*	Differential DNA methylation	Glycolysis, energy and lipid metabolism, adipogenesis and β cell function	[32]
	12 male and 12 female sibling pairs	Prenatal	∼58 y	*CPT1A* and *INSR*	Increased methylation	Growth and metabolism	[33]
	60 exposed; 60 sibling controls	Periconception	∼58 y ∼57 y	*IGF2/H19*	Differential DNA methylation	Growth and development	[34]
China	188 subjects in total	Prenatal and early postnatal life	∼54 y	*IGF2*	Increased methylation	Growth and development	[35]
	25 exposed subjects; 54 non-exposed controls	Prenatal	Late adulthood	*ENO2, ZNF226, CCDC51* and *TMA7*	Differential methylation	Cell proliferation, glycolysis and neurogenesis	[38]
	75 exposed subjects; 160 controls	Prenatal and early postnatal life	∼55 y	*INSR*	Increased methylation	Development, growth and metabolism	[39, 40]
Bangladesh	143	Prenatal, early postnatal life	Adult	*VTRNA2-1, PAX8, PRDM-9, ZFP57, BOLA* and *EXD3*	Differential DNA methylation	Development, growth and metabolism	[40]

Recently, evidence for the possibility of undernutrition-mediated epigenetic programming was also obtained in several Asian populations. For example, early-life exposure to the Chinese famine of 1959–61 was found to be related to elevated levels of methylation of *IGF2* gene and also total cholesterol in late adult life [37]. In a genome-wide DNA methylation analysis, differentially methylated regions associated with exposure to famine have been revealed in four genes (*ENO2, ZNF226, CCDC51* and *TMA7*), and pathways implicated in neurogenesis and development of nervous system were identified as the most significantly affected by developmental nutritional deprivation [38]. In people prenatally exposed to this famine, increased methylation level of *INSR* gene involved in growth and metabolism has been found in late adulthood [39]. More recently, prenatal Chinese famine exposure was found to be significantly associated with higher overall methylation level of *INSR* gene and larger adulthood waist circumference [40]. From these and other findings, it has been suggested that early-life exposure to the Chinese famine can be an important contributor to current epidemics of T2D in China [27, 41]. Regarding possible mechanisms, epigenetic changes may also be involved in increased odds of developing T2D in adulthood in individuals born during the famine in eastern Ukraine in the first 6 months of 1934 [25]. However, specific effects on gene expression need further investigation. In Bangladesh, significant differences in DNA methylation levels have been reported in seven metastable epialleles previously shown to vary with prenatal famine exposure [42]. However, due to insufficient sample size, the study of Finer and colleagues was not able to detect DNA methylation differences according to the developmental windows studied. Further studies are needed of gestational famine exposure that is accompanied by epigenetic differences at specific metastable epialleles. The authors concluded that exposure to this famine early in life caused epigenetic programming towards obesity and T2D in adult life. Prenatal exposure to famine was also shown to be able to induce epigenetic changes in pathways other than those involved in metabolic regulation. For example, significant hypermethylation in the promoter of the schizophrenia candidate gene, such as *DUSP22*, was observed in the Chinese famine-exposed schizophrenia patients [43]. There is convincing evidence about an increased risk of schizophrenia in adult life caused by prenatal exposure to famine. Indeed, a two-fold increased relative risk in the prenatally exposed vs the no exposed cohorts was demonstrated; moreover, these observations were similar to the Dutch studies [44]. The data of both China and Dutch studies showed that exposure to famine through early gestation is the critical period for increased risk of schizophrenia. Hence, similar findings of schizophrenia risk after prenatal exposure to famine in ethnically and culturally distinct populations of China and the Netherlands suggested about the same processes involved and may apply in all populations undergoing famine.

Interestingly, differential adult-life epigenetic signatures, in particular, differential methylation in gene pathways involved in cell cycle, development and apoptosis, were shown to be associated with birth seasonality [45]. It seems to be an important point because there is evidence that season of birth can influence adult health outcomes and even longevity [46, 47] and that adverse health outcomes can be more pronounced in those persons who were born in periods when the famine-induced food deprivation was further aggravated by the seasonally constrained food availability [46].

## Concluding Remarks

On the basis of information provided in the previous section, it can be concluded that epigenetic mechanisms may substantially contribute to long-term outcomes of developmental famine exposure. This seems all the more likely given that early gestation, a period when epigenome is most labile and susceptible to environmental stimuli [8], was found to be the most vulnerable stage in this respect [23, 48]. In addition, epigenetics seems to be the most plausible candidate mechanism for mediating these effects because experience-driven changes in epigenetic profiles may stably persist throughout life, thereby increasing the risk for chronic disorders via the mechanism of developmental programming. Of course, other factors, such as tissue and organ alterations, (re)programming of major endocrine axes, changes in gut microbiota composition and early-life immune maturation [2], as well as selective survival of embryos in conditions of severe maternal starvation [49], could also be in play. Moreover, all these changes (including epigenetic ones) could be induced by famine-associated factors other than starvation (e.g. stress and infections). Therefore, further research is certainly needed to elucidate the causative mechanisms underlying long-term effects of early-life famine exposure on human health status.

## Data Availability

Data might be available under request to corresponding author.
